# Lake water chemistry and population of origin interact to shape fecundity and growth in *Daphnia ambigua*

**DOI:** 10.1002/ece3.10176

**Published:** 2023-06-20

**Authors:** Mary A. Rogalski, Utku Ferah

**Affiliations:** ^1^ Bowdoin College Brunswick Maine USA

**Keywords:** calcium, common garden, *Daphnia ambigua*, ion availability, reciprocal transplant, salinity

## Abstract

Freshwater environments vary widely in ion availability, owing to both natural and anthropogenic drivers. Field and laboratory work point to the importance of overall salinity, as well as cation depletion, in shaping the physiology, behavior, and ecology of freshwater taxa. Yet, we currently have a poor understanding of the degree to which populations may vary in response to ion availability. Using *Daphnia* collected from three lakes that differ greatly in salinity and calcium availability, we conducted a laboratory reciprocal transplant experiment to assess how animals representing these populations vary in fecundity, body size, and survival when reared in lake water from each environment. The lake water environment and population of origin strongly interacted to shape *Daphnia* growth and reproduction. Surprisingly, we found only modest evidence that lake water with abundant calcium (5.5 vs. 1.2–2.3 mg/L) increased *Daphnia* growth or reproduction. By contrast, water from a relatively ion‐rich lake (400 μS/cm specific conductance) strongly boosted *Daphnia* fecundity over lower‐ion lake water (20–50 μS/cm), especially for the population originating from the high‐ion environment. Our results suggest that ion‐poor conditions common in regions around the world may exert stress on freshwater organisms, even for populations inhabiting these environments. Meanwhile, moderate salt enrichment may not prove harmful but could even benefit freshwater taxa in these ion‐poor regions. The context dependence of how and when lake water chemistry affects *Daphnia* and other freshwater taxa deserves greater attention, in both ion‐depleted and ion‐rich conditions. *Daphnia* are key members of lake food webs and serve as an important model for ecology, evolution, and toxicology research. Consideration of how lake water chemistry may influence how *Daphnia* populations respond to abiotic and biotic stress may improve the ability to evaluate and predict ecological and evolutionary dynamics in lakes of varying chemical composition.

## Graphical abstract

Freshwater environments differ widely in salt availability, yet we have little understanding of intraspecific variation in response to ion gradients in freshwater taxa. In our laboratory reciprocal transplant experiment, *Daphnia* fecundity greatly decreased in relatively ion‐poor conditions and calcium availability did little to mitigate this effect. Animals from a lake modestly enriched with sea salt were especially sensitive to the switch to lower‐ion conditions.

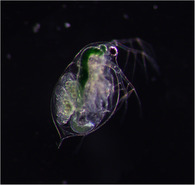

## 1 INTRODUCTION

Salt availability varies widely across freshwater environments around the world and is shaped by both natural and anthropogenic drivers. Human activities including application of road salt during winter storms, changes in precipitation and evaporation patterns, and sea level rise have led to widespread salinization of freshwater bodies (Dugan et al., 2017; Estévez et al., 2019; Kaushal et al., 2018). Meanwhile, deforestation and acidic deposition resulting from industrial activity have resulted in the depletion of some essential cations (e.g., calcium and magnesium) from surface waters (Hessen et al., 2017; Jeziorski et al., 2008; Kahl et al., 1991; Norton et al., 1989; Stoddard et al., 1999). These anthropogenic influences overlay natural gradients in ion availability driven by geology, climate, and proximity to the coast (Estévez et al., 2019; Huser et al., 2020; Norton et al., 1989). Major ions (Na^+^, Ca^2+^, Mg^2+^, K^+^, and Cl^−^) are essential micronutrients, needed for survival, growth, and reproduction (Kaspari, 2021). These ions play a key role in osmoregulation, as well as nervous system and motor system functioning, while calcium also serves as an important structural micronutrient (Bell et al., 1993; Cairns & Yan, 2009; Metz et al., 2014; Spence et al., 2012). At the same time, elevated concentrations of dissolved ions can be toxic to freshwater organisms (Brauner et al., 2012; Griffith, 2017). Determining the extent to which the ion landscape shapes ecological and evolutionary dynamics has emerged as a critical area of research (Kaspari, 2021).

Both ion‐poor and extremely ion‐rich freshwater environments are likely to impose physiological stress on freshwater biota. All freshwater organisms contend with the osmo‐ionoregulatory challenges of limiting water uptake and preventing ion loss to the surrounding environment. Most freshwater animals, including fish and crustaceans, are hyper‐osmoregulators (Griffith, 2017)—they maintain ion concentrations higher than the surrounding waters by actively pumping ions (mainly Na^+^ and Cl^−^) across permeable membranes, minimizing ion leakage, and excreting excess water. Osmoregulation in ion‐poor environments is metabolically costly (Glazier & Sparks, 1997), and this stress may be further exacerbated by aqueous calcium depletion, which increases the permeability of gill epithelia (Brauner et al., 2012; Gundersen & Curtis, 1995). Calcium also serves as a key structural micronutrient, important for building bone and the chitinous exoskeleton in crustaceans. Calcium limitation leads to increased mortality, reduced body size, slower maturation, and reduced reproductive output in a variety of crustacean taxa (Ashforth & Yan, 2008; Cairns & Yan, 2009; Hessen et al., 2000). Low‐calcium availability may also increase vulnerability to predation, for example, by limiting the formation of inducible defenses (Riessen et al., 2012) and reducing exoskeleton rigidity (Edwards et al., 2015). Exposure to elevated concentrations of dissolved ions can also prove stressful, with toxicity occurring when environmental concentrations approach or exceed that of intracellular fluids and hemolymph (Griffith, 2017). Thus, the cost of maintaining physiological homeostasis should shift across gradients of ion availability, with increased metabolic costs expected in freshwater environments at either extreme, and with calcium availability playing a particularly important role in supporting survival, growth, and reproduction.

Variation among taxa in the ability to tolerate ion availability likely plays a role in shaping community structure. Both ion‐poor and extremely ion‐rich freshwater environments tend to support lower diversity and altered species composition, with these trends observed across a range of taxa, including bacteria, zooplankton, mollusks, macroinvertebrates, and plants (Gutierrez et al., 2018; Piscart et al., 2005; Sowa et al., 2019). Field observations of shifting community structure along ion gradients may be difficult to disentangle from other abiotic or biotic variables that correlate with ion availability, such as pH, macronutrient availability, pollution, or changes in predation regime (Griffiths et al., 2019). Yet experimental studies support that ion availability per se can play an important role in shaping freshwater communities (Arnott et al., 2017; Lind et al., 2018; Sinclair & Arnott, 2018). In low‐ion environments, calcium availability appears to be an important determinant of zooplankton population dynamics and community structure (Arnott et al., 2017; Durant et al., 2018); species with higher calcium needs tend to be absent or rare, while species with low calcium needs may become more competitive and dominant (Durant et al., 2018; Edwards et al., 2015; Jeziorski et al., 2008, 2012, 2014; Wærvågen et al., 2002). Daphniids are especially vulnerable to calcium depletion (Cairns & Yan, 2009), likely owing to the need to frequently replace their relatively high calcium exoskeleton following each molt during their life cycle (Alstad et al., 1999); they rely primarily on aqueous calcium to meet these demands (Tan & Wang, 2009). At the other end of the salinity spectrum, while many species are lost, some salt‐tolerant species thrive in habitats naturally rich in ions (Michels et al., 2003; Ortega‐Mayagoitia et al., 2000). Such salt‐loving taxa have been observed colonizing environments experiencing anthropogenic salt inputs, even great distances from their typical geographic range (e.g., Hairston et al., 2005; Kipriyanova et al., 2007).

Given the significant physiological stress and ecological impacts associated with both ion‐poor and high‐salinity conditions, ion availability should exert selection pressure on freshwater taxa. Limited examination of intraspecific phenotypic variation among natural populations has provided some evidence of evolution in response to ion availability. Local adaptation to elevated salinity was observed in *Daphnia pulex* and *Simocephalus vetulus* populations inhabiting ponds impacted by sea spray or saltwater intrusion (Loureiro et al., 2012; Weider & Hebert, 1987). Experimental evolution work showed *Daphia pulex* can evolve tolerance for elevated sodium chloride (NaCl) in only 5–10 generations (Coldsnow et al., 2017). In addition, *Ambystoma maculatum* (spotted salamander) populations inhabiting roadside vernal pools showed evidence of local adaptation to road salt (Brady, 2012). Yet surprisingly *Lithobates sylvaticus* (wood frog) populations in the same wetlands displayed clear evidence of local *mal*adaptation to road salt stress (Brady, 2013). Ion‐poor freshwater environments also appear to drive a range of evolutionary responses. Low‐calcium conditions may select for reduced investment in pelvic size in three‐spined stickleback (*Gasterosteus aculeatus*) in the absence of strong predation pressure (Bell et al., 1993; Spence et al., 2012). In addition, *Daphnia pulicaria* from seven Canadian Shields lakes showed interclonal differences in response to calcium limitation but with little relation to the population of origin (Overhill, 2017). Meanwhile, *Daphnia galeata* from a low‐calcium lake were more sensitive to calcium restriction relative to those from a higher calcium environment (Alstad Rukke, 2002). This evidence supports that both ion‐poor and ion‐enriched environments may shape the evolutionary trajectories of freshwater organisms; however, the variation in responses among taxa, including both adaptive and maladaptive responses to ion availability, points to the need for further study.

**FIGURE 1:**
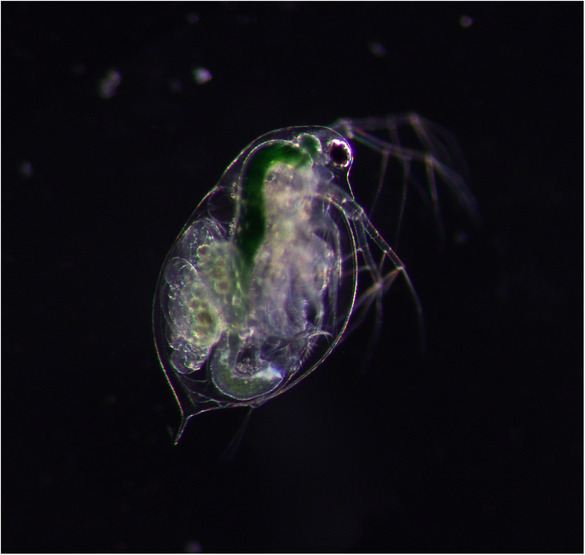
Image of a mature *Daphnia ambigua* from Hall Pond, reared in Sewall Pond water, with embryos developing in the brood chamber (credit: Mary Rogalski).

Here, we used a laboratory reciprocal transplant experiment to examine how *Daphnia ambigua* isolated from three natural populations differed in response to being reared in lake water with widely varying ion availability and composition: two inland lakes, one of which is especially ion‐poor, and a coastal lake where sea salt influence has caused both ion enrichment and a shift in ion composition (Figure 1). This design allowed us to determine the extent to which these varying lake water environments influenced *Daphnia* growth and reproduction and whether the population of origin interacted to shape these responses.

## 2 METHODS

### 2.1 Study lake selection

**FIGURE 2:**
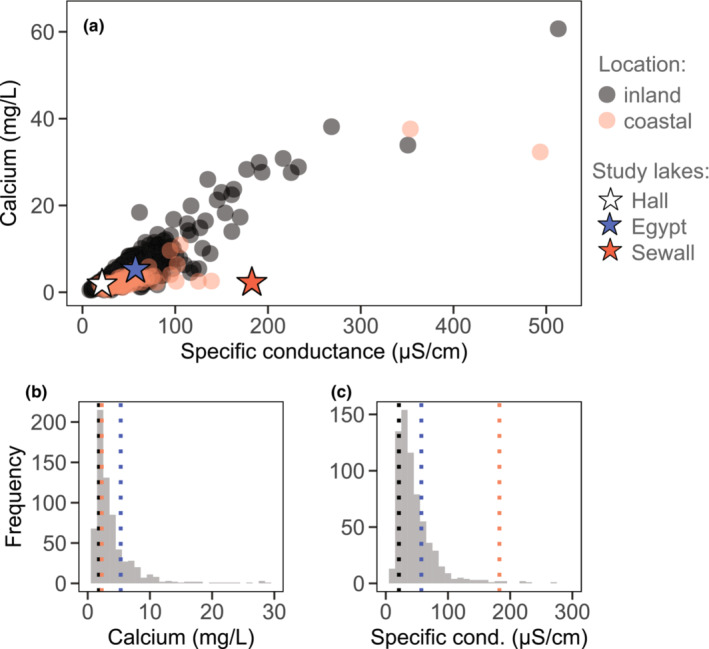
Variation in aqueous calcium concentrations and specific conductance among Maine lakes sampled by the Maine Department of Environmental Protection between 1996 and 2012. (a) Each data point represents the average concentrations measured for a given lake during this time (*N* = 688 lakes). Study lakes are marked with stars (white: Hall Pond, blue: Egypt Pond, orange: Sewall Pond). Histograms show frequency of calcium (b) and specific conductance (c) levels observed in this data set, with the study lakes noted with dotted vertical lines (black: Hall Pond, blue: Egypt Pond, orange: Sewall Pond).

Both natural and anthropogenic factors influence the ion chemistry of lakes in our study region of Maine, USA. Bedrock, soil properties, hydrology, and watershed activities influence the availability of important cations like calcium (Ca^2+^) and (Mg^2+^), while sodium (Na^+^) and chloride (Cl^−^) availability increases with proximity to the coast, as well as anthropogenic inputs in the watershed (Norton et al., 1989). Overall, ion availability in Maine lakes tends to be low relative to other lakes in the United States (National Lakes Assessment, 2007) (Figure S1). In addition, of the 688 lakes regularly sampled by the Maine Department of Environmental Protection (DEP) in the mid‐1990s‐early 2010s (unpublished data), about 10% have concentrations of Ca^2+^ below 1.5 mg/L (Figure 2), the minimum concentration thought to support *Daphnia pulicaria* in ideal laboratory conditions (Ashforth & Yan, 2008). Using this historic data collected by the Maine DEP, we selected three study lakes of similar size (17–29 hectares) and hydroperiod (permanent) that represent some of the breadth of this variation in ion availability and composition (Figure 2) and support populations of our focal species, *Daphnia ambigua*. Hall Pond, with a specific conductance in the 10th percentile, is ion‐poor even compared with other Maine lakes (Figure S1). By contrast, Sewall Pond is a relatively ion‐rich lake that receives sea salt from a brackish tidal creek (Figure 2a,c). Sewall Pond resembles other coastal Maine lakes (found within 10 km of the coast) in that Ca^2+^ tends to contribute relatively less to overall ion availability, while NaCl provides a greater contribution (Figure 2a, Figure S2). Finally, Egypt Pond has intermediate ion availability but with calcium levels far exceeding those in the other two study lakes (Figure 2b). The three study lakes are separated from one another by a geographic distance of at least 45 km (Figure S3).

### 2.2 Establishment of *Daphnia* clonal lineages

Our focal species, *Daphnia ambigua*, is a small *Daphnia* species with a widespread geographic distribution, found in lakes throughout North America, South America, and Europe, across a wide range of environmental gradients (Hebert et al., 2003). We established clonal cultures of *D. ambigua* from each study lake in late‐May 2019 from net collections (80‐μm mesh, 20‐cm diameter tow net, Wildco) sampling the full water column at the deepest basin of the lake. Zooplankton samples were kept in unfiltered lake water from the sampling site, in cool, dark conditions, and were processed within 2 h of collection. We examined the live zooplankton samples under a dissecting microscope at 10× magnification, isolating each female *D. ambigua* individual from random subsamples until at least 12 individuals were collected from each lake. Of the *Daphnia* that we collected that survived to reproduce, we randomly selected four clonal lineages from each lake to maintain for this study, using juveniles from the second to fifth brood to initiate each new generation.

### 2.3 Study lake characterization

In May, June, and July 2019 we sampled each lake to characterize its chemical and physical properties (Table 1) around the time of establishment of *Daphnia* clonal lineages. From the deepest basin of each lake, we collected a water sample at 1‐m depth using a Van Dorn bottle for water quality analyses. We measured anions (Cl^−^, NO_3_^−^, and SO_4_^−^) and cations (Na^+^, K^+^, Mg^2+^, and Ca^2+^) with a Dionex Ion Chromatograph ICS‐1100 (Thermo Fisher Corporation) with suppressed conductivity (Hautman et al., 1997). Total nitrogen and total phosphorus (TN and TP) were analyzed using alkaline persulfate digestion (Patton & Kryskalla, 2003). We measured chlorophyll *a* concentrations from an integrated water sample collected from the photic zone (the surface to twice the Secchi depth) in late‐May and early‐July 2019. Duplicate water subsamples were passed through glass fiber filters (Whatman GF/F, 0.7 μm pore size) and wet filters were frozen for 3 weeks before chlorophyll extraction (over 24 h in 10 mL 90% acetone at −20°C) and fluorometric analysis (Turner Designs Trilogy) (Holm‐Hansen & Riemann, 1978). Dissolved organic carbon (DOC) was measured by high temperature catalytic oxidation with a Shimadzu Total Organic Carbon analyzer (TOC‐VSH or TOC‐LSH; Shimadzu Corporation) from water samples collected in early June 2021; based on historic values, these DOC concentrations are likely to resemble levels in 2019 (Text S2).

**TABLE 1:** Chemical and physical characteristics of the three study lakes.

Lake	Date	Depth	Cond.	pH	Cl^−^	SO_4_^2−^	Na^+^	K^+^	Mg^2+^	Ca^2+^	DOC	Chl *a*	Secchi	TN	TP	*Z*_max_	SA
Hall	May 29, 2019^a^	1	18.2	6.83	3.11	0.56	2.04	0.21	0.23	1.24	—	1.31	6.6	0.20	32.02	7.9	20.6
	June 18, 2019^b^	1	—	—	3.25	0.56	2.05	0.21	0.24	1.27	—	—	—	0.20	7.52		
	June 27, 2019	1	21.1	7.02	2.66	0.53	2.56	0.22	0.19	1.07	—	3.82	5.9	0.26	14.18		
	June 27, 2019	7	24.2	5.80	2.76	0.56	2.69	0.24	0.26	1.28	—	—	—	0.46	27.53		
	June 3, 2021	1	20.0	—	5.11	0.65	2.96	0.25	0.36	1.37	3.35	—	5.6	0.26	7.70		
Egypt	May 16, 2019^a^	1	40.8	7.42	4.72	0.89	2.74	0.39	0.42	5.07	—	—	3.15	0.25	7.32	15.25	28.7
	May 28, 2019	1	44.7	7.38	4.78	1.02	2.74	0.45	0.47	5.26	—	4.79	3.8	0.17	—		
	June 18, 2019^b^	1	—	—	4.85	0.87	2.84	0.44	0.43	5.50	—	—	—	0.21	7.07		
	July 3, 2019	1	54.7	7.29	4.63	0.87	3.23	0.43	0.41	5.11	—	4.02	4.5	0.21	5.99		
	July 3, 2019	14	70.2	6.33	5.23	0.76	2.91	0.38	0.31	6.81	—	—	—	0.47	26.24		
	June 17, 2021	1	50.0	—	5.38	1.03	2.64	0.41	0.55	5.25	4.14	—	4.3	0.26	8.78		
Sewall	May 13, 2019^a^	1	435	5.98	117.56	4.97	65.15	2.98	6.06	3.03	—	—	1.9	0.26	39.14	3.7	17.4
	June 4, 2019	1	407.4	6.27	115.82	4.93	64.73	3.00	5.94	2.81	—	4.69	1.6	0.25	49.31		
	June 18, 2019^b^	1	—	—	111.87	4.67	62.06	2.86	5.84	2.80	—	—	—	0.25	8.12		
	July 11, 2019	1	384.8	6.55	105.93	2.53	57.65	2.01	5.53	1.81	—	7.9	1.25	0.40	26.71		
	July 11, 2019	2.5	—	—	109.50	4.56	53.46	2.51	5.51	2.63	—	—	—	0.43	32.32		
	May 31, 2021	1	420	—	104.69	4.86	60.10	2.35	6.71	3.43	5.14	—	0.75	0.27	13.57		

*Note*: Major ions, dissolved organic carbon (DOC), chlorophyll a (Chl a), and total nitrogen (TN) concentrations in mg/L; total phosphorus (TP) in μg/L; sampling depth (Depth), lake maximum depth (*Z*_max_), and Secchi depth (Secchi) in meters; surface area (SA) in hectares. pH and specific conductance (Cond.) (μS/cm) were measured in situ with a YSI‐ProDSS probe at the sampling depth indicated.

^a^ Indicates date when *Daphnia* clonal lineages were collected from each lake.

^b^ Indicates date lake water was collected for the reciprocal transplant trial.

### 2.4 *Daphnia* culturing conditions

Water for *Daphnia* culture maintenance was collected from each study lake in 20‐L polyethylene carboys, passed through glass fiber filters (Pall A/E, 1.0 μm pore size), and stored at 4°C before use. We maintained each *Daphnia* individual in a borosilicate beaker with 25 mL filtered water (warmed to room temperature) from their lake of origin. *Daphnia* cultures were maintained at 20°C with a 16‐h light:8‐h dark period. We changed the lake water and removed offspring twice weekly, and *Daphnia* were each fed 500,000 cells of *Ankistrodesmus falcatus* four times weekly. *Ankistrodesmus* was cultured in heat sterilized modified ASM‐1 medium (Goulden & Hornig, 1980) at room temperature, harvested weekly during the logistic growth phase, and stored at 4°C. A vitamin mixture (Goulden et al., 1982) was added to the algal culture to support *Daphnia* nutritional needs. Algal media and vitamin supplement constituents were reagent grade (Fisher Scientific). Our *Ankistrodesmus* strain originated from the lab of C. E. Goulden at the Academy of Natural Sciences in Philadelphia, PA, USA and has been designated the AJT strain (Schomaker & Dudycha, 2021). To ensure consistent feeding levels throughout the experiment and to minimize any effects of feeding on water chemistry, algae were settled and resuspended with filtered lake water, maintaining a density of 1,000,000 cells/mL using a hemocytometer. *Daphnia* were fed resuspended algae mixed with the same lake water used for their culturing.

### 2.5 Experimental design

To evaluate how *Daphnia* fitness is influenced by lake water chemistry and the extent to which *Daphnia* from the three populations varied in their response, we performed a laboratory reciprocal transplant experiment. Using four *Daphnia* clonal lineages collected from the three study lakes (low‐ion Hall Pond, high‐Ca^2+^ Egypt Pond, and ion‐rich Sewall Pond), we compared growth, reproduction, and survival over the course of 21 days, with genetically identical replicates reared in filtered lake water from one of each of the three study lakes.

Before conducting the experiment, *Daphnia* were reared in filtered lake water from their lake of origin for 3–5 generations in standard culturing conditions described above. We collected third to fifth brood *Daphnia* neonates aged 6–24 h from each clonal lineage to initiate the reciprocal transplant experiment. These *Daphnia* were transferred to 25 mL filtered lake water from either their home lake or one of the other two lakes. Otherwise, experimental animals were kept under the standard culturing conditions (20°C, 16:8 h light:dark, fed 500,000 cells *Ankistrodesmus falcatus* four times weekly, water changes twice weekly, in 25 mL of filtered lake water from the respective treatment). We included 10–12 replicate *Daphnia* individuals per lake × clonal lineage × lake water treatment in the experiment; however, difficulty maintaining two genotypes (one from Hall and one from Egypt Pond) and experimental error yielded 4–6 replicates per clonal lineage × treatment in a few cases (total number of replicates: 329).

Twice weekly we evaluated survival and counted and removed any offspring produced before moving the *Daphnia* to a clean beaker of filtered lake water. On the seventh day of the trial, when most *Daphnia* had reached maturity, we photographed each experimental animal under a dissecting microscope (Olympus SZX16, CelSens Entry imaging software) at 50× magnification. We measured body length from the center of the eye to the base of the tail‐spine using ImageJ. We ended the experiment after 21 days and collected a second set of body size measurements for each surviving experimental animal. While body size was not measured for neonates, these measurements of body length at days 7 and 21 allowed us to examine the influence of the lake water environment on investment in growth, when accounting for variation among populations and clones.

### 2.6 Statistical analyses

We evaluated whether the lake water environment, population of origin, or the interaction of these two variables predicted aspects of growth, reproduction, or survival using generalized linear mixed models (GLMMs). We selected the best fit model by comparing the Akaike information criterion (AIC) of the saturated model with nested simpler models containing fewer fixed effects. We used likelihood ratio tests to select which fixed effects to include in the final model, based on Zuur et al. (2009). We evaluated the normality of the residuals of the selected models using visual inspection of quantile plots and Kolmogorov–Smirnov tests.

Models examining variation in body size and growth included either body length at 7 days (around the timing of first reproduction for most animals), length at 21 days (the end of the trial), or the growth that occurred between days 7 and 21 ([size at 21 days − size at 7 days]/size at 7 days) as the response variable. We applied an arcsine square root transformation of the proportion growth to improve the normality of the residuals. To assess investment in reproduction, we examined whether reproduction had occurred by the 7th day of the trial (binomial response variable) and the total number offspring produced during the 21‐day trial. *Daphnia* that died before day 7 of the trial (*N* = 10 of 329 replicates) were excluded from analyses of growth and reproduction. In a separate GLMM, we evaluated whether the likelihood of survival to maturity (as a binomial variable) was predicted by water source, population of origin, or their interaction.

In each GLMM, clonal lineage was included as a random intercept. All analyses were conducted using the statistical program R (v. 4.2.1) (R Core Team, 2016). Mixed effects modeling was conducted using the “glmmTMB” package (Brooks et al., 2017). Differences in growth, reproduction, and survival among all pairwise lake and treatment comparisons were evaluated using post hoc tests of Tukey's Honest Significant Difference (HSD) with the “emmeans” package.

## 3 RESULTS

### 3.1 Body size

**FIGURE 3:**
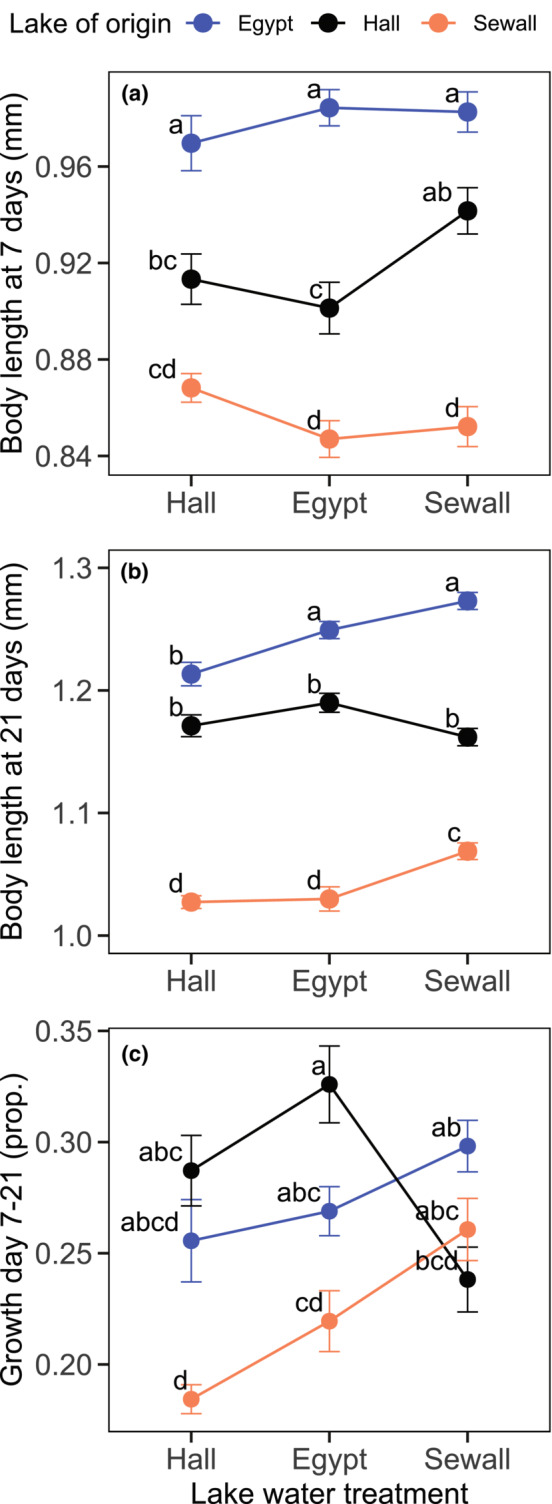
Body size and growth rate varied depending on lake of origin, the lake water environment, and their interaction. (a) 7‐day mean body size of *Daphnia ambigua* clonal replicates from the three study populations (Hall Pond = low‐ion, Egypt Pond = high‐Ca^2+^, Sewall Pond = ion‐rich) reared in lake water collected from each study lake. (b) Final (21‐day) mean body size of the same individuals. (c) Proportion increase in body size that occurred over the final 2 weeks of the experiment ([21‐day size − 7‐day size]/7‐day size). Error bars represent ±1 standard error of the mean across individuals from all clones (*N* = 30–40 total replicates from four clones/lake). Lake water environment is shown on the *x* axis and colors represent the population of origin. ‘a–d’ designate significant pairwise differences (*p* < .05) among groups in post hoc Tukey HSD tests of GLMM results, where the same letter indicates no significant difference.

Seven days into the trial, *Daphnia* body length was strongly influenced by lake of origin (*p* < .001, *χ* ^2^ = 32.854 AIC = ‐958.70, Figure 3a). *Daphnia* from Egypt Pond (high‐Ca^2+^ lake) were 6.7% larger than those from Hall Pond (low‐ion lake) (Tukey HSD, *p* < .001, *t* ratio = 5.340), which were 7.4% larger than animals from Sewall Pond (ion‐rich lake) (Tukey HSD, *p* < .001, *t* statistic = 5.589). The lake water environment did not significantly affect body size measured on day 7 (*p* = .115, *χ* ^2^ = 4.330, AIC = −959.03). There was a significant interaction between lake of origin and treatment (*p* = .013, *χ* ^2^ = 12.623, AIC = −963.65); however, we observed no significant pairwise differences in size when comparing body length on day 7 in the natal and transplant lake water environments for any of the populations (Figure 3a).

By the end of the trial (21 days), *Daphnia* body size was strongly impacted by lake of origin (*p* < .001, *χ* ^2^ = 33.826, AIC = −1025.58), lake water treatment (*p* < .001, *χ* ^2^ = 23.500, AIC = −1045.08), and the interaction between origin and treatment (*p* < .001, *χ* ^2^ = 39.635, AIC = −1076.71). Overall, Sewall Pond *Daphnia* were about 15% smaller than the animals from the other two lakes (Figure 3b) but grew to a larger size in their natal lake water environment compared with when reared in water from either the high‐Ca^2+^ (Tukey HSD, *p* = .002, *t* ratio = −4.138) or low‐ion (Tukey HSD, *p* < .001, *t* ratio = −4.518) lakes. *Daphnia* from high‐Ca^2+^ Egypt Pond also grew to a larger size in their home lake water compared with their growth in the low‐Ca^2+^ water from Hall Pond (Tukey HSD, *p* = .009, *t* ratio = 3.653); however, Egypt Pond *Daphnia* reached a similar size in the ion‐rich conditions of Sewall Pond's water (Tukey HSD, *p* = .319, *t* ratio = −2.344). Hall Pond *Daphnia* body length did not differ when comparing size in their home and transplant lake water environments (Tukey HSDs, Hall vs. Egypt water: *p* = .499, *t* ratio = 2.065; Hall vs. Sewall water: *p* = 0.985, *t* ratio = 1.005).

The growth that occurred over the last 2 weeks of the trial, during the primary reproductive period, was associated with lake of origin (*p* = .010, *χ* ^2^ = 9.138, AIC = −564.81), lake water treatment (*p* = .025, *χ* ^2^ = 7.412, AIC = −568.22), and the interaction between origin and treatment (*p* < .001, *χ* ^2^ = 38.576, AIC = −598.80). Sewall Pond *Daphnia* grew at a faster rate in their home lake water environment compared with the low‐ion (Hall Pond) water treatment (Tukey HSD, *p* < .001, *t* ratio = −4.461), while *Daphnia* from Hall and Egypt Ponds grew at a similar rate when comparing growth in their home lake water environment and the transplant treatments (Figure 3c).

### 3.2 Reproduction

**FIGURE 4:**
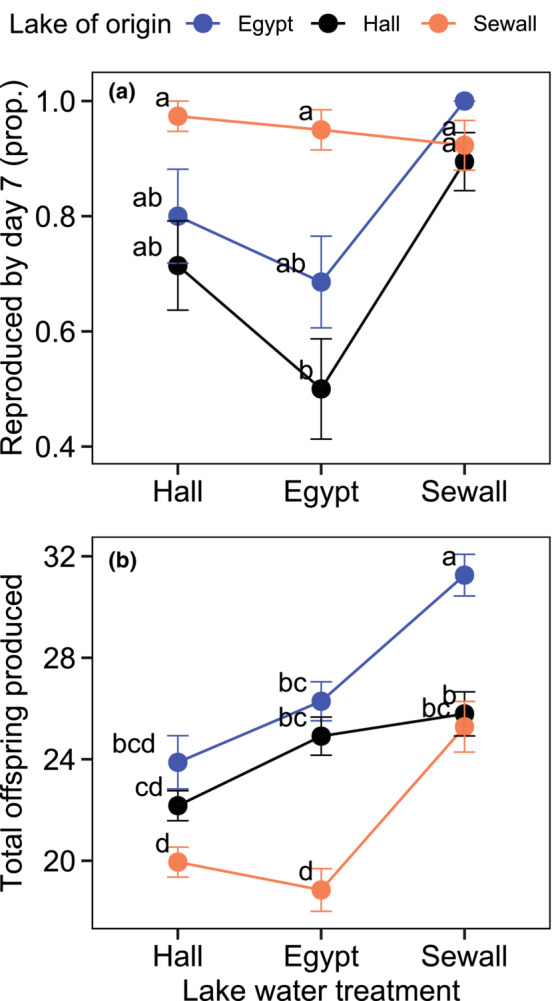
Timing of first reproduction and overall fecundity were influenced by the interaction of lake of origin and lake water treatment. Hall Pond = low‐ion, Egypt Pond = high‐Ca^2+^, Sewall Pond = ion‐rich. (a) Proportion of *Daphnia* replicates that reproduced by the seventh day of the trial. (b) Total offspring produced over the course of the 21‐day experiment. Error bars represent ±1 standard error of the mean across replicates from all clones (*N* = 30–40 total replicates from four clones/lake). Lake water environment is shown on the *x* axis and colors represent the population of origin. ‘a–d’ designates significant pairwise differences (*p* < .05) among groups in post hoc Tukey tests of GLMM results, where the same letter indicates no significant difference. Note: In panel A, Egypt *Daphnia* in Sewall water had 100% separation (no replicates delayed reproduction beyond day 7), making it statistically complex to differentiate this group from the other eight population × treatment combinations.

The proportion of *Daphnia* that reproduced by the seventh day ranged from 36% to 100% across clones and treatments. This tendency to reproduce early in the trial varied according to population of origin (*p* = <.001, *χ* ^2^ = 24.641, AIC = 268.28), treatment (*p* < .001, *χ* ^2^ = 21.594, AIC = 250.69) and their interaction (*p* = .024, *χ* ^2^ = 11.222, AIC = 247.46; Figure 4a). Overall, *Daphnia* from Sewall Pond were more likely to reproduce early compared with animals from Hall Pond (Tukey HSD, *p* < .001, *t* ratio = −3.631). However, these differences diminished in the Sewall Pond water treatment, where *Daphnia* from all three lakes tended to reproduce by the seventh day (Figure 4a).

The total number of offspring produced over the 21‐day trial was strongly associated with population of origin (*p* = .002, *χ* ^2^ = 12.604, AIC = 1982.5, Figure 4b), lake water treatment (*p* < .001, *χ* ^2^ = 69.508, AIC = 1917.0), and the interaction between origin and treatment (*p* < .001, *χ* ^2^ = 18.698, AIC = 1906.3). Overall, *Daphnia* originating from Egypt Pond tended to produce more offspring than those from Sewall Pond (Tukey HSD, *p* < .001, *t* ratio = 3.811) and reproduction tended to be highest in Sewall Pond water compared with Hall (Tukey HSD, *p* < .001, *t* ratio = −8.676) or Egypt Pond water (Tukey HSD, *p* < .001, *t* ratio = −6.600). While *Daphnia* from Sewall Pond and Egypt Pond produced more offspring in Sewall Pond water compared with Egypt Pond water (34% more for Sewall *Daphnia*, Tukey HSD, *p* < .001; 19% more for Egypt *Daphnia*, Tukey HSD, *p* < .001), *Daphnia* from Hall Pond performed equally in these two environments (Tukey HSD, *p* = .997, *t* ratio = −0.775). Total offspring production in the Hall and Egypt Pond water treatments was no different for any of the *Daphnia* populations (Tukey HSDs: Hall *Daphnia*: *p* = .224; Egypt *Daphnia*: *p* = .436; Sewall *Daphnia*: *p* = .993).

### 3.3 Survival to maturity

**FIGURE 5:**
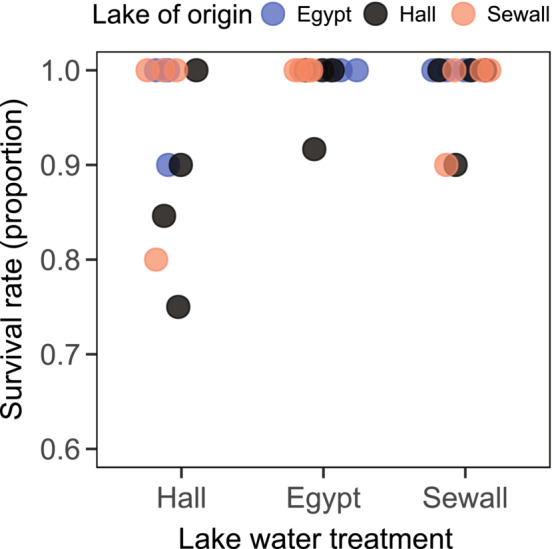
Rates of survival to maturity for *Daphnia* from the three study populations (Hall Pond = low‐ion, Egypt Pond = high‐Ca^2+^, Sewall Pond = ion‐rich) reared in lake water collected from each study lake. Each data point represents the proportion of *Daphnia* that survived to reproduce for each clonal lineage × treatment combination. Data points are slightly offset to increase visibility.

Early survival rates were high, with 97% of the 329 replicates surviving to maturity (Figure 5). There was modest support for lake water treatment explaining this variation (*p* = .037, *χ* ^2^ = 6.5983, AIC = 88.964), with seven of the 10 deaths occurring in Hall Pond water. However, post hoc Tukey tests showed no differences in survival rates among the lake water environments (Tukey HSD, *p*‐values from .139 to .851), suggesting limited power to evaluate pairwise differences among treatments. Lake of origin and the interaction between origin and treatment were not significant predictors of survival (origin: *p* = .222, *χ* ^2^ = 3.005, AIC = 89.959; origin × treatment: *p* = .723, *χ* ^2^ = 2.072, AIC = 95.887).

## 4 DISCUSSION

Lake water chemistry and population of origin strongly interacted to shape *Daphnia* growth and reproduction in this laboratory reciprocal transplant experiment. The impact of the lake water environment on reproduction was dramatic, with mean differences in overall fecundity as large as 34% depending on transplant conditions for a given population. Surprisingly, Egypt Pond's water with its elevated calcium levels provided little boost to fecundity for *Daphnia* from any of the three populations compared with Hall Pond's low‐ion environment. Meanwhile, Sewall Pond's ion‐rich environment showed no sign of reducing growth, reproduction or survival compared with the two lower‐ion environments. Rather, *Daphnia* from all three lakes produced more offspring in Sewall Pond water than the low‐ion Hall Pond environment. While we saw no evidence that *Daphnia* from high‐Ca^2+^ Egypt Pond were especially sensitive to Hall Pond's low‐ion conditions, we did see evidence that *Daphnia* from ion‐rich Sewall Pond experienced a relatively larger fecundity decrease when reared in both lower‐ion conditions. Together, this suggests that modest increases in salinity owing to coastal influence may support increased *Daphnia* fitness relative to low‐ion conditions commonly observed in Maine lakes, and populations may differ in the degree to which they respond to this variation in lake water chemistry.

A body of research drawing upon both field and laboratory study supports that calcium availability plays a critical role in supporting robust populations of *Daphnia* and other crustaceans with high calcium demands (Arnott et al., 2017; Ashforth & Yan, 2008; Cairns & Yan, 2009; Hessen et al., 2000; Jeziorski et al., 2012). Calcium may serve as a limiting structural micronutrient for *Daphnia* (Ashforth & Yan, 2008; Hessen et al., 2000), and low aqueous calcium is expected to increase the metabolic demands of osmoregulation in low‐ion freshwaters (Brauner et al., 2012). However, we observed little benefit to *Daphnia ambigua* growth or reproduction from Egypt Pond's high‐Ca^2+^ lake water environment. Laboratory studies have shown that *Daphnia* sp. tend to see a reduction in fitness between calcium levels of 0–2 mg/L, with levels below 1–0.5 mg/L lethal (Cairns & Yan, 2009). Above these critical thresholds, suboptimal calcium environments have more subtle effects on development, reproduction, calcification, and longevity (Alstad et al., 1999; Ashforth & Yan, 2008; Hessen et al., 2000). Though *D. ambigua* has received relatively little attention in explorations of calcium demands in *Daphnia*, recent work revealed that *D. ambigua* maintain relatively low hemolymph Ca^2+^ levels and are able to uptake Ca^2+^ at very low environmental concentrations compared with *D. pulicaria* and *D. magna* (Durant et al., 2018). This may explain the modest benefits observed in Egypt Pond's water (5.5 mg/L Ca^2+^) compared with water from Hall Pond (1.2 mg/L Ca^2+^). Our study controlled for other stressors *Daphnia* may experience in their natural environment, such as food limitation, elevated temperatures, predation, and disease, some of which have been shown to compound the challenges of calcium limitation (Ashforth & Yan, 2008; Celis‐Salgado et al., 2016; Huang et al., 2021; Pérez‐Fuentetaja & Goodberry, 2016; Riessen et al., 2012). Future research examining interactions between calcium limiting conditions and other stressors may find that even subtle effects on fitness caused by suboptimal calcium are ecologically relevant.

By contrast, lake water from ion‐rich Sewall Pond provided a measurable fecundity benefit for *Daphnia* from all three lakes. *Daphnia* from both Hall Pond and Egypt Pond produced more offspring in Sewall Pond water compared with their natal lower‐ion lake water environments, and animals from Sewall Pond were much less fecund and smaller in size when reared in water from the two lower‐ion lakes. In addition, *Daphnia* reared in Sewall Pond water were more likely to reproduce by the seventh day of the trial. This suggests that Sewall Pond's water provides a benefit that water from the two lower‐ion lakes lacks. While calcium concentrations are moderate in Sewall Pond, magnesium, potassium, and sodium are orders of magnitude higher than levels in both lower‐ion lakes. These salts or some other unmeasured micronutrient may be limiting in the lower‐ion lake waters. In addition, the metabolic costs of osmoregulation may be reduced in Sewall Pond's higher salinity water. Salt toxicity may not be expected to occur until ion levels approach or exceed concentrations in the hemolymph (Griffith, 2017), and ion levels in Sewall Pond (Table 1) are below concentrations measured in hemolymph of *Daphnia ambigua* reared experimentally in low‐ion conditions (Durant et al., 2018). In addition, dissolved organic carbon concentrations in Sewall Pond's water exceed levels found in the other two study lakes (Table 1). Experimental work suggests that lake browning may support increased survival and reproduction in *Daphnia longispina* (Minguez et al., 2020), though browning agents extracted from leonardite may poorly replicate the effects of naturally derived organic carbon on lake organisms (Scharnweber et al., 2021). Some combination of these factors, or another unmeasured difference in Sewall Pond's water may benefit *Daphnia* fitness over the low‐ion lake water environments. Research on the impacts of salt enrichment in freshwater environments has often focused on the stress caused by very high salinity resulting from sea salt or road salt pollution (Brady, 2012, 2013; Weider & Hebert, 1987). Our results suggest that in regions where ion‐poor conditions dominate, moderate introduction of sea salt may not prove harmful and could even benefit the resident freshwater taxa.

A central aim of this study was to explore not only the extent to which lake water chemistry influences *Daphnia* growth and reproduction but also the degree to which populations from divergent environments might vary in their responses. Overall, the environment that was most supportive of *Daphnia* growth and reproduction was Sewall Pond's ion‐rich water, and *Daphnia* originating from Sewall Pond were most strongly impacted by the stress of the lower‐ion environments. This evidence supports that *Daphnia* from Sewall Pond are adapted to their local lake water chemistry. By the end of the 21‐day trial, Sewall Pond *Daphnia* were larger and much more fecund in their natal lake water compared with low‐ion lake waters from Hall or Egypt Ponds (27%–34% increase in total offspring). *Daphnia* from the two lower‐ion lakes also showed increased fecundity in Sewall Pond's high‐ion environment compared with their natal lake water, but the differences were more modest (17%–19% increase). Meanwhile, we see no evidence that *Daphnia* from Egypt and Hall Pond are adapted to differences in their natal lake water. Both populations showed a similar pattern of a modest (nonsignificant) increase in fecundity in Egypt Pond's high‐Ca^2+^ environment. It is possible that if we allowed for multiple generations of acclimation, the study animals would find their transplant conditions more stressful (low‐Ca^2+^) or beneficial (high‐Ca^2+^), increasing the differences observed in contrasting calcium environments (Giardini et al., 2015). However, findings from a subsequent trial also showed a modest fecundity benefit of Egypt Pond's higher‐Ca^2+^ environment after a generation of acclimation, with no difference seen between the two populations (Figure S5). Future work examining the relative influence of genetics versus transgenerational plasticity may help elucidate mechanisms behind divergent population responses to alterations in water chemistry.

While we were careful to ensure that only the source of lake water and *Daphnia* clones differed among our experimental treatments, our study was not designed to manipulate specific aspects of lake water chemistry to evaluate their effects on *Daphnia* performance. This is both a limitation and a strength of our design. While we are unable to say what precisely makes Sewall Pond's water so beneficial to *Daphnia* reproduction or Egypt Pond's water less beneficial than expected, we were able to take a first step in examining the extent to which *Daphnia* from natural populations respond to divergent water chemistry and the extent to which they may be locally adapted to these differences. We are aware of only two other studies that explored adaptation to the whole lake water environment in *Daphnia* (Allen et al., 2010; Declerck et al., 2001). Both studies focused on the effects of resource availability (i.e., food quality and quantity), and neither study reported water chemistry parameters other than macronutrient availability (e.g., nitrogen and phosphorus), making their findings difficult to compare with ours. Yet these initial studies, like ours, indicate that *Daphnia* from different populations can respond dramatically to differences in lake water conditions, sometimes in unexpected ways. Further exploration of the influence of lake water representing a broader range of conditions will aid in our understanding of the degree to which *Daphnia* populations are locally adapted to their natal water chemistry. This work will generate hypotheses to further test empirically, strengthening our ability to understand the characteristics of water chemistry that most strongly influence freshwater organisms.

*Daphnia* are a model system for ecology, evolution, and toxicology research. A great body of research examines *Daphnia* responses to individual stressors associated with lake water chemistry and pollution using artificial lake water as a test medium (e.g., COMBO [Kilham et al., 1998], ADaM [Klüttgen et al., 1994], Elendt M7 [Samel et al., 1999], FLAMES [Celis‐Salgado et al., 2008]). The value in this approach lies in the ability of researchers to identify causal factors responsible for any observed differences among treatments. However, if *Daphnia* are sensitive to differences (whether measured or unknown) between their natal lake water and the artificial medium, the findings generated from these assays may not provide a fair assessment of how organisms embedded in their natural environment may be impacted by the variable of interest. Lake water chemistry may also strongly influence responses to other stressors, including predation (Bell et al., 1993; Huang et al., 2021; Riessen et al., 2012; Spence et al., 2012), parasitism (Buss & Hua, 2018; Milotic et al., 2017; Stockwell et al., 2015), temperature (Ashforth & Yan, 2008), toxicant pollution (Celis‐Salgado et al., 2016), and food limitation (Pérez‐Fuentetaja & Goodberry, 2016). Thus, researchers examining how *Daphnia* respond and adapt to abiotic and biotic stress should take care to consider how lake water chemistry and the choice of experimental test conditions may influence these responses.

Overall, our study supports that variation in ion availability and composition may strongly influence *Daphnia* growth and reproduction and that populations may vary in the strength of this response. The context dependence of how and when lake water chemistry affects *Daphnia* deserves greater attention, in both ion‐depleted environments and ion‐rich conditions. Individual ions may be important limiting micronutrients, but ion ratios and overall ion strength may also play a critical role in supporting *Daphnia* health or protecting *Daphnia* from toxic conditions (Celis‐Salgado et al., 2016; Davies & Hall, 2007; Elphick et al., 2011). At the same time, the source of ion enrichment is likely to strongly influence the degree to which added salts might be beneficial, as activities such as road salt application, agriculture, mining, and wastewater disposal are likely to be accompanied by harmful toxicants and may involve complex biogeochemical interactions (Kaushal et al., 2022). *Daphnia* serve as an important model system for ecology, evolutionary biology, and toxicology research, while also playing a critical role in lake food webs as regulators of phytoplankton productivity and food for fish and larger invertebrates (Lampert, 2011; Miner et al., 2012). Our findings suggest that accounting for the effects of natural variation in lake water chemistry may help to uncover previously unrecognized patterns in *Daphnia* ecology and evolution.

## AUTHOR CONTRIBUTIONS

**Mary A. Rogalski:** Conceptualization (lead); data curation (lead); formal analysis (lead); funding acquisition (lead); investigation (lead); methodology (lead); project administration (lead); resources (lead); software (lead); supervision (lead); validation (lead); visualization (lead); writing – original draft (lead); writing – review and editing (lead). **Utku Ferah:** Conceptualization (supporting); formal analysis (supporting); funding acquisition (supporting); investigation (supporting); project administration (supporting); writing – original draft (supporting); writing – review and editing (supporting).

## FUNDING INFORMATION

This study was supported by Bowdoin College and the Freedman Summer Research Fellowship in Coastal/Environmental Studies.

## CONFLICT OF INTEREST STATEMENT

None.

## Supporting information


Appendix S1.

## Data Availability

The data that support the findings of this study are openly available in Dryad at https://doi.org/10.5061/dryad.n5tb2rc06.

## References

[ece310176-bib-0001] Allen, M. R. , Thum, R. A. , & Cáceres, C. E. (2010). Does local adaptation to resources explain genetic differentiation among *Daphnia populations*? Molecular Ecology, 19(15), 3076–3087. 10.1111/j.1365-294X.2010.04728.x 20609079

[ece310176-bib-0002] Alstad, N. , Skardal, L. , & Hessen, D. (1999). The effect of calcium concentration on the calcification of *Daphnia magna* . Limnology and Oceanography, 44(8), 2011–2017. 10.1152/ajplegacy.1945.145.1.67

[ece310176-bib-0003] Alstad Rukke, N. (2002). Tolerance to low ambient calcium shows inter‐population differences in *Daphnia galeata* . Journal of Plankton Research, 24(5), 527–531.

[ece310176-bib-0004] Arnott, S. E. , Azan, S. , & Ross, A. (2017). Calcium decline reduces population growth rates of zooplankton in field mesocosms. Canadian Journal of Zoology, 95, 323–333.

[ece310176-bib-0005] Ashforth, D. , & Yan, N. D. (2008). The interactive effects of calcium concentration and temperature on the survival of *Daphnia pulex* at high and low food concentrations reproduction. Limnology and Oceanography, 53(2), 420–432.

[ece310176-bib-0006] Bell, M. A. , Orti, G. , Walker, J. A. , & Koenings, J. P. (1993). Evolution of pelvic reduction in threespine stickleback fish: A test of competing hypotheses. Evolution, 47(3), 906. 10.2307/2410193 28567888

[ece310176-bib-0007] Brady, S. P. (2012). Road to evolution? Local adaptation to road adjacency in an amphibian (*Ambystoma maculatum*). Scientific Reports, 2, 235. 10.1038/srep00235 22355748 PMC3267261

[ece310176-bib-0008] Brady, S. P. (2013). Microgeographic maladaptive performance and deme depression in response to roads and runoff. PeerJ, 1, e163. 10.7717/peerj.163 24109548 PMC3792186

[ece310176-bib-0009] Brauner, C. J. , Gonzalez, R. J. , & Wilson, J. M. (2012). Extreme environments: Hypersaline, alkaline, and ion‐poor waters. Fish Physiology, 32, 435–476. 10.1016/B978-0-12-396951-4.00009-8

[ece310176-bib-0010] Brooks, M. , Kristensen, K. , van Benthem, K. , Magnusson, A. , Berg, C. , Nielsen, A. , Skaug, H. J. , Mächler, M. , & Bolker, B. (2017). glmmTMB balances speed and flexibility among packages for zero‐inflated generalized linear mixed modeling. The R Journal, 9, 378–400.

[ece310176-bib-0011] Buss, N. , & Hua, J. (2018). Parasite susceptibility in an amphibian host is modified by salinization and predators. Environmental Pollution, 236, 754–763. 10.1016/j.envpol.2018.01.060 29455088

[ece310176-bib-0012] Cairns, A. , & Yan, N. D. (2009). A review of the influence of low ambient calcium concentrations on freshwater daphniids, gammarids, and crayfish. Environmental Review, 17, 67–79.

[ece310176-bib-0013] Celis‐Salgado, M. P. , Cairns, A. , Kim, N. , & Yan, N. D. (2008). The FLAMES medium: A new, soft‐water culture and bioassay medium for Cladocera. SIL Proceedings, 1922–2010, 30(2), 265–271. 10.1080/03680770.2008.11902123

[ece310176-bib-0014] Celis‐Salgado, M. P. , Keller, W. , & Yan, N. D. (2016). Calcium and sodium as regulators of the recovery of four *Daphnia* species along a gradient of metals and base cations in metal contaminated lakes in Sudbury, Ontario, Canada. Journal of Limnology, 75, 36–49. 10.4081/jlimnol.2016.1271

[ece310176-bib-0015] Coldsnow, K. D. , Mattes, B. M. , Hintz, W. D. , & Relyea, R. A. (2017). Rapid evolution of tolerance to road salt in zooplankton. Environmental Pollution, 222, 367–373. 10.1016/j.envpol.2016.12.024 28065573

[ece310176-bib-0016] Davies, T. D. , & Hall, K. J. (2007). Importance of calcium in modifying the acute toxicity of sodium sulphate to *Hyalella azteca* and *Daphnia magna* . Environmental Toxicology and Chemistry, 26(6), 1243–1247. 10.1897/06-510R.1 17571691

[ece310176-bib-0017] Declerck, S. , Cousyn, C. , & De Meester, L. (2001). Evidence for local adaptation in neighbouring *Daphnia* populations: A laboratory transplant experiment. Freshwater Biology, 46(2), 187–198. 10.1046/j.1365-2427.2001.00632.x

[ece310176-bib-0018] Dugan, H. A. , Bartlett, S. L. , Burke, S. M. , Doubek, J. P. , Krivak‐Tetley, F. E. , Skaff, N. K. , Summers, J. C. , Farrell, K. J. , McCullough, I. M. , Morales‐Williams, A. M. , Roberts, D. C. , Ouyang, Z. , Scordo, F. , Hanson, P. C. , & Weathers, K. C. (2017). Salting our freshwater lakes. Proceedings of the National Academy of Sciences of the United States of America, 114(17), 4453–4458. 10.1073/pnas.1620211114 28396392 PMC5410852

[ece310176-bib-0019] Durant, A. C. , Celis‐Salgado, M. P. , Ezatollahpour, S. , Yan, N. D. , Arnott, S. E. , & Donini, A. (2018). Ca2+ levels in *Daphnia* hemolymph may explain occurrences of daphniid species along recent Ca gradients in Canadian soft‐water lakes. Comparative Biochemistry and Physiology Part A: Molecular and Integrative Physiology, 218, 8–15. 10.1016/j.cbpa.2018.01.009 29366920

[ece310176-bib-0020] Edwards, B. A. , Jackson, D. A. , & Somers, K. M. (2015). Evaluating the effect of lake calcium concentration on the acquisition of carapace calcium by freshwater crayfish. Hydrobiologia, 744(1), 91–100. 10.1007/s10750-014-2059-2

[ece310176-bib-0021] Elphick, J. R. F. , Bergh, K. D. , & Bailey, H. C. (2011). Chronic toxicity of chloride to freshwater species: Effects of hardness and implications for water quality guidelines. Environmental Toxicology and Chemistry, 30(1), 239–246. 10.1002/etc.365 20872898

[ece310176-bib-0022] Estévez, E. , Rodríguez‐Castillo, T. , González‐Ferreras, A. M. , Cañedo‐Argüelles, M. , & Barquín, J. (2019). Drivers of spatio‐temporal patterns of salinity in Spanish rivers: A nationwide assessment. Philosophical Transactions of the Royal Society, B: Biological Sciences, 374(1764), 1–10. 10.1098/rstb.2018.0022 PMC6283964 30509921

[ece310176-bib-0023] Giardini, J.‐L. , Yan, N. D. , & Heyland, A. (2015). Consequences of calcium decline on the embryogenesis and life history of *Daphnia magna* . Journal of Experimental Biology, 218(13), 2005–2014. 10.1242/jeb.123513 25944923

[ece310176-bib-0024] Glazier, D. S. , & Sparks, B. L. (1997). Energetics of amphipods in ion‐poor waters: Stress resistance is not invariably linked to low metabolic rates. Functional Ecology, 11(1), 126–128. 10.1046/j.1365-2435.1997.00061.x

[ece310176-bib-0025] Goulden, C. E. , Commotto, R. M. , Hendrickson, J. A. J. , Hornig, L. L. , & Johnson, K. L. (1982). Procedures and recommendations for the culture and use of *Daphnia* in bioassay studies. *Aquatic Toxicology and Hazard Assessment: Proceedings of the Fifth Annual Symposium on Aquatic Toxicology: A Symposium*, 139–160.

[ece310176-bib-0026] Goulden, C. E. , & Hornig, L. L. (1980). Population oscillations and energy reserves in planktonic cladocera and their consequences to competition. Proceedings of the National Academy of Sciences of the United States of America, 77(3), 1716–1720. 10.1073/pnas.77.3.1716 16592788 PMC348568

[ece310176-bib-0027] Griffith, M. B. (2017). Toxicological perspective on the osmoregulation and ionoregulation physiology of major ions by freshwater animals: Teleost fish, crustacea, aquatic insects, and Mollusca. Environmental Toxicology and Chemistry, 36(3), 576–600. 10.1002/etc.3676 27808448 PMC6114146

[ece310176-bib-0028] Griffiths, K. , Winegardner, A. K. , Beisner, B. E. , & Gregory‐Eaves, I. (2019). Cladoceran assemblage changes across the Eastern United States as recorded in the sediments from the 2007 National Lakes Assessment, USA. Ecological Indicators, 96, 368–382. 10.1016/j.ecolind.2018.08.061

[ece310176-bib-0029] Gundersen, D. T. , & Curtis, L. R. (1995). Acclimation to hard or soft water at weakly alkaline pH influences gill permeability and gill surface calcium binding in rainbow trout (*Oncorhynchus mykiss*). Canadian Journal of Fisheries and Aquatic Sciences, 52(12), 2583–2593. 10.1139/f95-848

[ece310176-bib-0030] Gutierrez, M. F. , Tavşanoğlu, Ü. N. , Vidal, N. , Yu, J. , Teixeira‐de Mello, F. , Çakiroglu, A. I. , He, H. , Liu, Z. , Jeppesen, E. , & Jeppesen, E. (2018). Salinity shapes zooplankton communities and functional diversity and has complex effects on size structure in lakes. Hydrobiologia, 813(1), 237–255. 10.1007/s10750-018-3529-8

[ece310176-bib-0031] Hairston, N. G. , Kearns, C. M. , Demma, L. P. , & Effler, S. W. (2005). Species‐specific *Daphnia* phenotypes: A history of industrial pollution and pelagic ecosystem response. Ecology, 86(7), 1669–1678. 10.1890/03-0784

[ece310176-bib-0032] Hautman, D. P. , Munch, D. J. , & Pfaff, J. D. (1997). US EPA method 300.1: Determination of inorganic anions in drinking water by ion chromatography. US Environmental Protection Agency.

[ece310176-bib-0033] Hebert, P. D. N. , Witt, J. D. S. , & Adamowicz, S. J. (2003). Phylogeographical patterning in *Daphnia ambigua*: Regional divergence and intercontinental cohesion. Limnology and Oceanography, 48(1 I), 261–268. 10.4319/lo.2003.48.1.0261

[ece310176-bib-0034] Hessen, D. O. , Alstad, N. E. W. , & Skardal, L. (2000). Calcium limitation in Daphnia magna. Journal of Plankton Research, 22(3), 553–568.

[ece310176-bib-0035] Hessen, D. O. , Andersen, T. , Tominaga, K. , & Finstad, A. G. (2017). When soft waters becomes softer; drivers of critically low levels of Ca in Norwegian lakes. Limnology and Oceanography, 62(1), 289–298. 10.1002/lno.10394

[ece310176-bib-0036] Holm‐Hansen, O. , & Riemann, B. (1978). Chlorophyll a determination : Improvements in methodology. Oikos, 30(3), 438–447. 10.2307/3543338

[ece310176-bib-0037] Huang, J. , Xu, X. , Li, D. , Sun, Y. , Gu, L. , Zhang, L. , Lyu, K. , & Yang, Z. (2021). Decreased calcium concentration interferes with life history defense strategies of *Ceriodaphnia cornuta* in response to fish kairomone. Limnology and Oceanography, 66(8), 3237–3252. 10.1002/lno.11876

[ece310176-bib-0038] Huser, B. J. , Futter, M. N. , Bogan, D. , Brittain, J. E. , Culp, J. M. , Goedkoop, W. , Gribovskaya, I. , Karlsson, J. , Lau, D. C. P. , Rühland, K. M. , Schartau, A. K. , Shaftel, R. , Smol, J. P. , Vrede, T. , & Lento, J. (2020). Spatial and temporal variation in Arctic freshwater chemistry—Reflecting climate‐induced landscape alterations and a changing template for biodiversity. Freshwater Biology, 67(1), 14–29. 10.1111/fwb.13645

[ece310176-bib-0039] Jeziorski, A. , Paterson, A. M. , & Smol, J. P. (2012). Crustacean zooplankton sedimentary remains from calcium‐poor lakes: Complex responses to threshold concentrations. Aquatic Sciences, 74(1), 121–131. 10.1007/s00027-011-0202-y

[ece310176-bib-0040] Jeziorski, A. , Tanentzap, A. J. , Yan, N. D. , Paterson, A. M. , Palmer, M. E. , Korosi, J. B. , Rusak, J. A. , Arts, M. T. , Keller, W. B. , Ingram, R. , Cairns, A. , & Smol, J. P. (2014). The jellification of north temperate lakes. Proceedings of the Royal Society B: Biological Sciences, 282(1798), 20142449. 10.1098/rspb.2014.2449 PMC4262185 25411451

[ece310176-bib-0041] Jeziorski, A. , Yan, N. D. , Paterson, A. M. , DeSellas, A. M. , Turner, M. A. , Jeffries, D. S. , Keller, B. , Weeber, R. C. , McNicol, D. K. , Palmer, M. E. , McIver, K. , Arseneau, K. , Ginn, B. K. , Cumming, B. F. , & Smol, J. P. (2008). The widespread threat of calcium decline in fresh waters. Science, 322(5906), 1374–1377. 10.1126/science.1164949 19039134

[ece310176-bib-0042] Kahl, J. S. , Norton, S. A. , Cronan, C. S. , Fernandez, I. J. , Bacon, L. C. , & Haines, T. A. (1991). Maine. In D. F. Charles (Ed.), Acidic deposition and aquatic ecosystems: Regional case studies (pp. 203–235). Springer‐Verlag Inc.

[ece310176-bib-0043] Kaspari, M. (2021). The invisible hand of the periodic table: How micronutrients shape ecology. Annual Review of Ecology, Evolution, and Systematics, 52(1), 199–219. 10.1146/annurev-ecolsys-012021-090118

[ece310176-bib-0044] Kaushal, S. S. , Likens, G. E. , Pace, M. L. , Utz, R. M. , Haq, S. , Gorman, J. , & Grese, M. (2018). Freshwater salinization syndrome on a continental scale. Proceedings of the National Academy of Sciences of the United States of America, 115(4), E574–E583. 10.1073/pnas.1711234115 29311318 PMC5789913

[ece310176-bib-0045] Kaushal, S. S. , Mayer, P. M. , Likens, G. E. , Reimer, J. E. , Maas, C. M. , Rippy, M. A. , Grant, S. B. , Hart, I. , Utz, R. M. , Shatkay, R. R. , Wessel, B. M. , Maietta, C. E. , Pace, M. L. , Duan, S. , Boger, W. L. , Yaculak, A. M. , Galella, J. G. , Wood, K. L. , Morel, C. J. , … Becker, W. D. (2022). Five state factors control progressive stages of freshwater salinization syndrome. Limnology and Oceanography Letters, 8, 190–211. 10.1002/lol2.10248 PMC10395323 37539375

[ece310176-bib-0046] Kilham, S. S. , Kreeger, D. A. , Lynn, S. G. , Goulden, C. E. , & Herrera, L. (1998). COMBO: A defined freshwater culture medium for algae and zooplankton. Hydrobiologia, 377, 147–159. 10.1023/A:1003231628456

[ece310176-bib-0047] Kipriyanova, L. M. , Yermolaeva, N. I. , Bezmaternykh, D. M. , Dvurechenskaya, S. Y. , & Mitrofanova, E. Y. (2007). Changes in the biota of Chany Lake along a salinity gradient. Hydrobiologia, 576(1), 83–93. 10.1007/s10750-006-0295-9

[ece310176-bib-0048] Klüttgen, B. , Dülmer, U. , Engels, M. , & Ratte, H. T. (1994). ADaM, an artificial freshwater for the culture of zooplankton. Water Research, 28(3), 743–746. 10.1016/0043-1354(94)90157-0

[ece310176-bib-0049] Lampert, W. (2011). Daphnia: Development of a model organism in ecology and evolution. In O. Kinne (Ed.), Excellence in ecology series. International Ecology Institute.

[ece310176-bib-0050] Lind, L. , Schuler, M. S. , Hintz, W. D. , Stoler, A. B. , Jones, D. K. , Mattes, B. M. , & Relyea, R. A. (2018). Salty fertile lakes: How salinization and eutrophication alter the structure of freshwater communities. Ecosphere, 9(9), e02383. 10.1002/ecs2.2383

[ece310176-bib-0051] Loureiro, C. , Castro, B. B. , Claro, M. T. , Alves, A. , Arminda Pedrosa, M. , & Gonçalves, F. (2012). Genetic variability in the tolerance of natural populations of *Simocephalus vetulus* (Müller, 1776) to lethal levels of sodium chloride. Annales de Limnologie – International Journal of Limnology, 48(1), 95–103. 10.1051/limn/2012002

[ece310176-bib-0052] Metz, J. R. , Leeuwis, R. H. J. , Zethof, J. , & Flik, G. (2014). Zebrafish (*Danio rerio*) in calcium‐poor water mobilise calcium and phosphorus from scales. Journal of Applied Ichthyology, 30(4), 671–677. 10.1111/jai.12513

[ece310176-bib-0053] Michels, E. , Audenaert, E. , Ortells, R. , & De Meester, L. (2003). Population genetic structure of three pond‐inhabiting *Daphnia* species on a regional scale (Flanders, Belgium). Freshwater Biology, 48(10), 1825–1839. 10.1046/j.1365-2427.2003.01132.x

[ece310176-bib-0054] Milotic, D. , Milotic, M. , & Koprivnikar, J. (2017). Effects of road salt on larval amphibian susceptibility to parasitism through behavior and immunocompetence. Aquatic Toxicology, 189, 42–49. 10.1016/j.aquatox.2017.05.015 28582700

[ece310176-bib-0055] Miner, B. E. , De Meester, L. , Pfrender, M. E. , Lampert, W. , & Hairston, N. G. J. (2012). Linking genes to communities and ecosystems: *Daphnia* as an ecogenomic model. Proceedings of the Royal Society B: Biological Sciences, 279, 1873–1882. 10.1098/rspb.2011.2404 PMC3311900 22298849

[ece310176-bib-0056] Minguez, L. , Sperfeld, E. , Berger, S. A. , Nejstgaard, J. C. , & Gessner, M. O. (2020). Changes in food characteristics reveal indirect effects of lake browning on zooplankton performance. Limnology and Oceanography, 65(5), 1028–1040. 10.1002/lno.11367

[ece310176-bib-0057] National Lakes Assessment . (2012). Retrieved from U.S. Environmental Protection Agency website. https://www.epa.gov/national‐aquatic‐resource‐surveys/nla

[ece310176-bib-0058] Norton, S. A. , Brakke, D. F. , Kahl, J. S. , & Haines, T. A. (1989). Major influences on lake water chemistry in Maine. Maine Geological Survey, 5, 109–124.

[ece310176-bib-0059] Ortega‐Mayagoitia, E. , Armengol, X. , & Rojo, C. (2000). Structure and dynamics of zooplankton in a semi‐arid wetland, the National Park Las Tablas de Daimiel (Spain). Wetlands, 20(4), 629–638. 10.1672/0277-5212(2000)020[0629:SADOZI]2.0.CO;2

[ece310176-bib-0060] Overhill, M. (2017). Intra‐specific variation in sensitivity to low calcium in *Daphnia pulex* (Master of Science, Queens University). Master of Science, Queens University. https://qspace.library.queensu.ca/handle/1974/22630

[ece310176-bib-0061] Patton, C. J. , & Kryskalla, J. R. (2003). Methods of analysis by the U.S. Geological Survey National Water Quality Laboratory—Evaluation of alkaline persulfate digestion as an alternative to Kjeldahl digestion for determination of total and dissolved nitrogen and phosphorus in water. In U.S. Geological Survey, *Water‐Resources Investigations Report 03‐4174*. Denver, CO.

[ece310176-bib-0062] Pérez‐Fuentetaja, A. , & Goodberry, F. (2016). *Daphnia*'s challenge: Survival and reproduction when calcium and food are limiting. Journal of Plankton Research, 38, 1379–1388. 10.1093/plankt/fbw077

[ece310176-bib-0063] Piscart, C. , Moreteau, J. C. , & Beisel, J. N. (2005). Biodiversity and structure of macroinvertebrate communities along a small permanent salinity gradient (Meurthe River, France). Hydrobiologia, 551(1), 227–236. 10.1007/s10750-005-4463-0

[ece310176-bib-0064] R Core Team . (2016). R: A language and environment for statistical computing (No. 3.2.4). R Foundation for Statistical Computing https://www.r‐project.org/

[ece310176-bib-0065] Riessen, H. P. , Linley, R. D. , Altshuler, I. , Rabus, M. , Sollradl, T. , Clausen‐Schaumann, H. , Laforsch, C. , & Yan, N. D. (2012). Changes in water chemistry can disable plankton prey defenses. Proceedings of the National Academy of Sciences of the United States of America, 109(38), 15377–15382. 10.1073/pnas.1209938109 22949653 PMC3458369

[ece310176-bib-0066] Samel, A. , Ziegenfuss, M. , Goulden, C. E. , Banks, S. , & Baer, K. N. (1999). Culturing and bioassay testing of *Daphnia magna* using Elendt M4, Elendt M7, and COMBO media. Ecotoxicology and Environmental Safety, 43(1), 103–110. 10.1006/eesa.1999.1777 10330328

[ece310176-bib-0067] Scharnweber, K. , Peura, S. , Attermeyer, K. , Bertilsson, S. , Bolender, L. , Buck, M. , Einarsdóttir, K. , Garcia, S. L. , Gollnisch, R. , Grasset, C. , Groeneveld, M. , Hawkes, J. A. , Lindström, E. S. , Manthey, C. , Övergaard, R. , Rengefors, K. , Sedano‐Núñez, V. T. , Tranvik, L. J. , & Székely, A. J. (2021). Comprehensive analysis of chemical and biological problems associated with browning agents used in aquatic studies. Limnology and Oceanography: Methods, 19(12), 818–835. 10.1002/lom3.10463

[ece310176-bib-0068] Schomaker, R. A. , & Dudycha, J. L. (2021). De novo transcriptome assembly of the green alga *Ankistrodesmus falcatus* . PLoS One, 16, 1–14. 10.1371/journal.pone.0251668 PMC8121315 33989339

[ece310176-bib-0069] Sinclair, J. S. , & Arnott, S. E. (2018). Local context and connectivity determine the response of zooplankton communities to salt contamination. Freshwater Biology, 63(10), 1273–1286. 10.1111/fwb.13132

[ece310176-bib-0070] Sowa, A. , Krodkiewska, M. , Halabowski, D. , & Lewin, I. (2019). Response of the mollusc communities to environmental factors along an anthropogenic salinity gradient. Science of Nature, 106(11–12), 60. 10.1007/s00114-019-1655-4 31758263

[ece310176-bib-0071] Spence, R. , Wootton, R. J. , Przybylski, M. , Zieba, G. , MacDonald, K. , & Smith, C. (2012). Calcium and salinity as selective factors in plate morph evolution of the three‐spined stickleback (*Gasterosteus aculeatus*). Journal of Evolutionary Biology, 25(10), 1965–1974. 10.1111/j.1420-9101.2012.02585.x 22862551

[ece310176-bib-0072] Stockwell, M. P. , Clulow, J. , & Mahony, M. J. (2015). Evidence of a salt refuge: Chytrid infection loads are suppressed in hosts exposed to salt. Oecologia, 177(3), 901–910. 10.1007/s00442-014-3157-6 25416999

[ece310176-bib-0073] Stoddard, J. L. , Jeffries, D. S. , Lükewille, A. , Clair, T. A. , Dillon, P. J. , Driscoll, C. T. , Forsius, M. , Johannessen, M. , Kahl, J. S. , Kellogg, J. H. , Kemp, A. , Mannio, J. , Monteith, D. T. , Murdoch, P. S. , Patrick, S. , Rebsdorf, A. , Skjelkvåle, B. L. , Stainton, M. P. , Traaen, T. , … Wllander, A. (1999). Regional trends in aquatic recovery from acidification in North America and Europe. Nature, 401(6753), 575–578. 10.1038/44114

[ece310176-bib-0074] Tan, Q.‐G. , & Wang, W.‐X. (2009). The regulation of calcium in *Daphnia magna* reared in different calcium environments. Limnology and Oceanography, 54(3), 746–756. 10.4319/lo.2009.54.3.0746

[ece310176-bib-0075] Wærvågen, S. B. , Rukke, N. A. , & Hessen, D. O. (2002). Calcium content of crustacean zooplankton and its potential role in species distribution. Freshwater Biology, 47(10), 1866–1878. 10.1046/j.1365-2427.2002.00934.x

[ece310176-bib-0076] Weider, L. J. , & Hebert, P. D. N. (1987). Ecological and physiological differentiation among low‐arctic clones of *Daphnia pulex* . Ecology, 68(1), 188–198.

[ece310176-bib-0077] Zuur, A. F. , Ieno, E. N. , Walker, N. J. , Saveliev, A. A. , & Smith, G. M. (2009). Mixed effects models and extensions in ecology with R. Springer. 10.4324/9780429201271-2

