# Supplementary material for: Lake water chemistry and population of origin interact to shape fecundity and growth in Daphnia ambigua
Source: Ecol Evol. 2023 Jun 20;13(6):e10176. doi: 10.1002/ece3.10176 (PMC10282168; doi:10.1002/ece3.10176)
Supplement: Supplementary file 1 — Appendix S1. [file ECE3-13-e10176-s001.docx]

**Appendix**

**Text S1. Ion chemistry of Maine lakes**

A comparison of lakes sampled in the US Environmental Protection Agency’s National Lakes Assessment in 2007 (“National Lakes Assessment,” 2012) and 688 lakes sampled by the Maine Department of Environmental Protection (DEP) between 1996-2012 (unpublished) shows that the specific conductance (a measure of total ionic charge) of Maine lakes tends to fall well below typical values observed in the rest of the country (Figure S1).

**
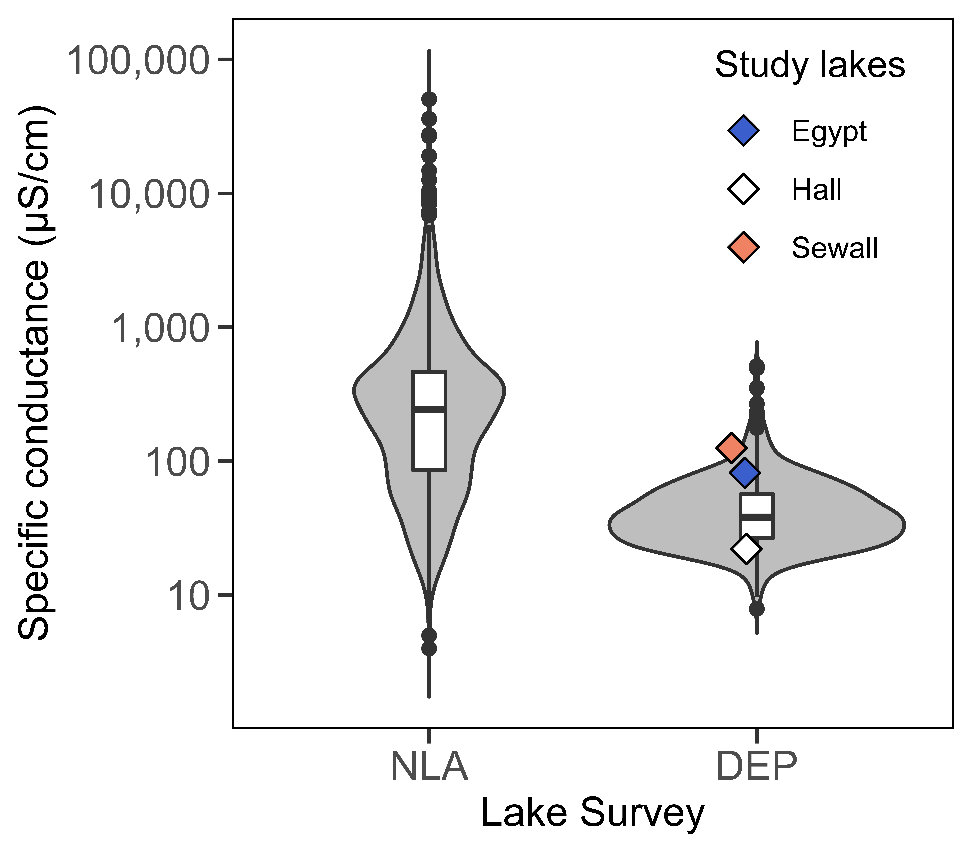
**

**Figure S1. Maine lakes tend to have low ion availability relative to those in the rest of the United States.** NLA shows data collected for the US Environmental Protection Agency’s National Lakes Assessment (NLA) in 2007 (N=1157); DEP shows specific conductance for 688 lakes sampled by the Maine Department of Environmental Protection (DEP) between 1996-2012. Diamonds represent mean specific conductance for lakes in this study, measured during the Maine DEP sampling period. Study lake points are jittered horizontally to better visualize the quantiles in the DEP box plot. Note the y axis is plotted on a log_10_ scale.

The relative contribution of various major ions to specific conductance varies geographically among Maine lakes. A principal components analysis (PCA) of Maine DEP data shows that lakes found within 10 km of the coast tend to have higher concentrations of sodium and chloride, and lower concentrations of calcium and magnesium for a given specific conductance, reflecting this sea salt influence (Figure S2). Sewall Pond, one of the most ion-rich lakes sampled in the survey, follows the trajectory of these coastal lakes, with high loadings on the PC1 axis and low values on the PC2 axis, due to its high specific conductance and relatively high sodium and chloride values.

**
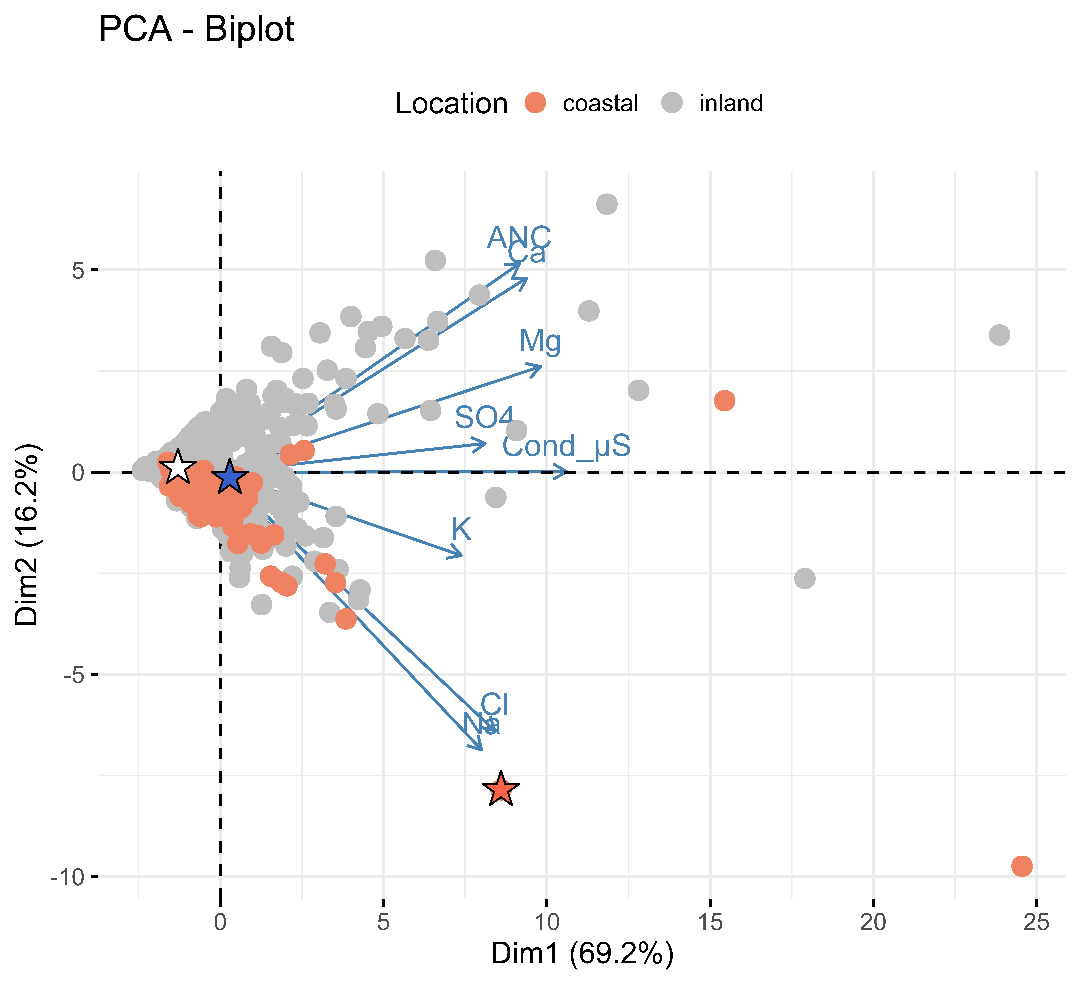
**

**Figure S2.** Biplot of the first two PC axes of a principal components analysis (PCA) of ion availability data collected by the Maine Department of Environmental Protection between 1996-2012. Each data point represents mean values for each lake over this time period (N=676 lakes). Lakes located within 10 km of the coast are shown orange. Study lakes are designated with stars (Hall=white, Egypt=blue, Sewall=orange). PCA input variables include concentrations of the major ions (Ca^2+^, Mg^2+^, K^+^, SO_4_^-^, Na^+^, and Cl^-^), anion neutralizing capacity (ANC), and specific conductance (Cond_µS).

**Reference:**

National Lakes Assessment. United States Environmental Protection Agency. 2007. URL: https://www.epa.gov/national-aquatic-resource-surveys/nla


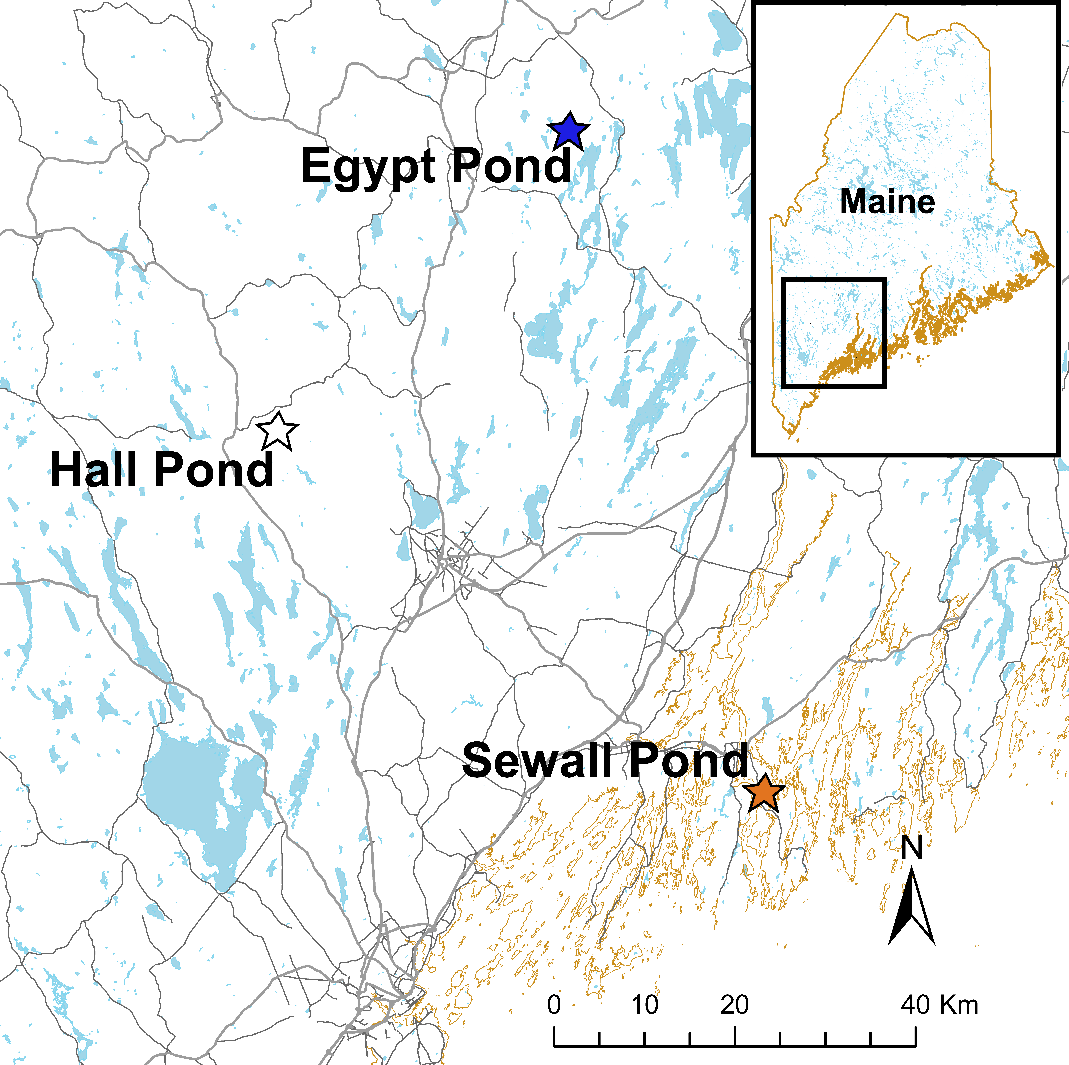


**Figure S3.** Map showing the study region in mid-coast Maine. All lakes and ponds with permanent hydroperiod are shown in light blue, with the three study lakes marked with stars. Tan lines represent the coast, and grey lines designate major and minor highways.

**Text S2.** Samples collected to analyze dissolved organic carbon (DOC) in summer 2019 were unable to be run due to complications related to the COVID-19 pandemic. We were able to analyze DOC concentrations from water samples collected in June 2021. Historic DOC levels in Hall Pond and Egypt Pond were very similar to the concentrations we measured in 2021 (Table S1). DOC concentrations in Sewall Pond have fluctuated more strongly and are negatively correlated with chloride concentrations (Figure S2). Based on this relationship, we expect that DOC levels in 2021 were similar to concentrations in 2019 since chloride levels were similar in summer of 2019 and 2021 (105 mg/L and 115 mg/L respectively).

**Table S1.** Dissolved organic carbon concentrations measured in surface water (1 m depth) in the study lakes. * indicates samples collected as part of this study. Previous data was collected by the Maine Department of Environmental Protection.

| **Lake** | **Date** | **DOC (mg/L)** |
| --- | --- | --- |
| Hall Pond | 21-Aug-1997 | 3.28 |
|  | 11-Aug-1998 | 4.0 |
|  | 9-Aug-2011 | 3.55 |
|  | 3-Jun-2021* | 3.35 |
| Egypt Pond | 13-Aug-1999 | 5.0 |
|  | 17-Jun-2021* | 4.14 |
| Sewall Pond | 24-Aug-1998 | 6.21 |
|  | 13-Aug-1999 | 7.6 |
|  | 1-Sep-2005 | 7.61 |
|  | 18-Aug-2008 | 7.45 |
|  | 31-May-2021* | 5.14 |


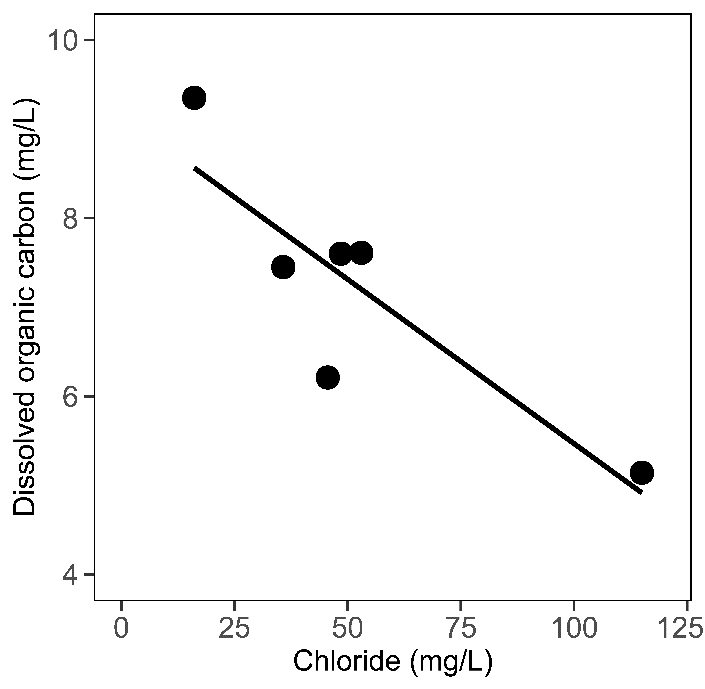


**Figure S4**. Dissolved organic carbon concentrations in Sewall Pond tend to decrease with increasing concentrations of chloride.


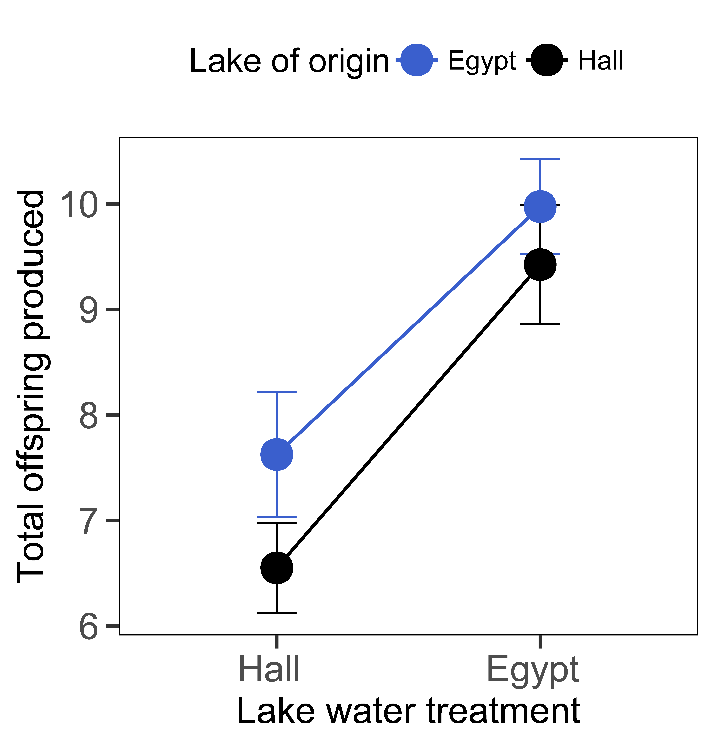


**Figure S5.** During a 14-day trial, where *Daphnia* were allowed one generation to acclimate to test waters, *D*. *ambigua* from Hall Pond and Egypt Pond produced about 2.5 more offspring in Egypt Pond water compared with low-Ca^2+^ Hall Pond water. This trial was conducted in August 2020, using the same clonal lineages that were tested in the main text above. Data points represent mean offspring produced over the 14-day trial for *Daphnia* from Egypt Pond (blue) or Hall Pond (black) reared in lake water from one of the two lakes (x-axis). The same clonal lineages used in the main text were included in this trial (N=4 clonal lineages/ lake, with 10 replicates per clone/treatment). Calcium concentrations were slightly higher in both lakes in 2020 (Hall Pond: 1.8 mg/L and Egypt Pond 7.1 mg/L).
